# Intramedullary nailing of femoral shaft fractures in polytraumatized patients. a longitudinal, prospective and observational study of the procedure-related impact on cardiopulmonary- and inflammatory responses

**DOI:** 10.1186/1757-7241-20-2

**Published:** 2012-01-05

**Authors:** Elisabeth E Husebye, Torstein Lyberg, Helge Opdahl, Trude Aspelin, Ragnhild Ø Støen, Jan Erik Madsen, Olav Røise

**Affiliations:** 1 Department of Orthopedics, Oslo University Hospital, Ullevaal, Norway; 2 Center for Clinical Research, Oslo University Hospital, Ullevaal, Norway; 3 The Norwegian National Center for NBC Medicine, Oslo University Hospital, Ullevaal, Norway; 4 Faculty of Medicine, University of Oslo, Norway; 5 Division of Emergencies and Critical Care, Oslo University Hospital, Norway

**Keywords:** intramedullary reaming, intramedullary nailing, inflammatory response, femoral shaft fracture, long bone fracture, polytrauma, coagulation and fibrinolysis, cytokine activation, cardiopulmonary response to trauma

## Abstract

**Background:**

Early intramedullary nailing (IMN) of long bone fractures in severely injured patients has been evaluated as beneficial, but has also been associated with increased inflammation, multi organ failure (MOF) and morbidity. This study was initiated to evaluate the impact of primary femoral IMN on coagulation-, fibrinolysis-, inflammatory- and cardiopulmonary responses in polytraumatized patients.

**Methods:**

Twelve adult polytraumatized patients with femoral shaft fractures were included. Serial blood samples were collected to evaluate coagulation-, fibrinolytic-, and cytokine activation in arterial blood. A flow-directed pulmonary artery (PA) catheter was inserted prior to IMN. Cardiopulmonary function parameters were recorded peri- and postoperatively. The clinical course of the patients and complications were monitored and recorded daily.

**Results:**

Mean Injury Severity Score (ISS) was 31 ± 2.6. No procedure-related effect of the primary IMN on coagulation- and fibrinolysis activation was evident. Tumor necrosis factor alpha (TNF-α) increased significantly from 6 hours post procedure to peak levels on the third postoperative day. Interleukin-6 (IL-6) increased from the first to the third postoperative day. Interleukin-10 (IL-10) peaked on the first postoperative day. A procedure-related transient hemodynamic response was observed on indexed pulmonary vascular resistance (PVRI) two hours post procedure. 11/12 patients developed systemic inflammatory response syndrome (SIRS), 7/12 pneumonia, 3/12 acute lung injury (ALI), 3/12 adult respiratory distress syndrome (ARDS), 3/12 sepsis, 0/12 wound infection.

**Conclusion:**

In the polytraumatized patients with femoral shaft fractures operated with primary IMN we observed a substantial response related to the initial trauma. We could not demonstrate any major additional IMN-related impact on the inflammatory responses or on the cardiopulmonary function parameters. These results have to be interpreted carefully due to the relatively few patients included.

**Trial Registration:**

ClinicalTrials.gov: NCT00981877

## Background

The appropriate timing of internal stabilization of long bone fractures has been and still seems to be controversial [1-5]. Early fracture stabilization has, in several studies, been associated with reduced pulmonary complications and mortality [6-11], whereas in other studies early intramedullary nailing (IMN) has been associated with increased inflammation, multi organ failure (MOF) and morbidity [12-14]. Severe trauma results in a generalized inflammatory response that can pose a threat to the organism. This response results in vasodilatation with leakage of fluids from the blood vessels, aggregation of platelets and leukocytes with clogging of small capillaries and dysfunction of involved organs. The lungs are most frequently affected, with function failure spanning from mild dysfunction to grave failure (adult respiratory distress syndrome (ARDS)). In patients with severe chest trauma Pape et al. found a higher incidence of posttraumatic ARDS and mortality when early femoral IMN was done. In trauma patients, operative procedures, such as IMN, represent a second insult, and the ideal time for such treatment, to pose as little harm as possible, has been a topic of discussion [6,15]. The aim of the study was to evaluate the additional procedure-related impact of primary femoral IMN on coagulation-, fibrinolysis-, inflammatory- and cardiopulmonary responses in polytraumatized patients.

## Methods

### Material

This study was approved by the Regional Committee for Medical and Health Research (ref. 02066). Informed consent or assent was obtained from the patients or their representative prior to inclusion in this study. In cases when the patients were unable to give personal consent at hospital admission, the personal consent was given posterity.

Inclusion criteria were patients, 18-65 years of age, admitted to the hospital between May 2003 and December 2004 with femoral diaphyseal fracture suitable for initial IMN within 24 hours post injury. The exclusion criteria were previous femoral shaft fracture, pathological fracture, femoral deformities or pregnancy.

The injury severity was assessed according to the Abbreviated Injury Scale (AIS) and scored by a certified AIS-registrar [16,17]. The overall severity of the injuries was calculated according to the Injury Severity Score (ISS) and New Injury Severity Score (NISS) [18,19]. The time from injury to hospital admittance and to IMN, AO classification, Gustilo Anderson classification of open fractures, and amount of blood products transfused were recorded. The clinical course of the patients and complications were monitored and recorded daily. Number of days in the intensive care unit (ICU) was also noted. Severely injured patients were defined as patients with ISS ≥ 16 [20,21]. The definitions of systemic inflammatory response syndrome (SIRS) and sepsis were according to the Consensus Conference of the American College of Chest Physicians and the Society of Critical Care Medicine of 1992 [22]. The definitions of acute lung injury (ALI) and ARDS were according to the American-European Consensus Conference on Acute Respiratory Distress Syndrome of 1994 [23].

### Cardiopulmonary monitoring

An 18-gauge arterial line was placed in the radial artery in all patients for continuous recording of arterial pressures. A flow-directed pulmonary artery (PA) catheter (744HF75, Swan-Ganz CCOmbo CCO/SvO_2 _catheter 7.5F, Edwards Critical-Care Division, Irvine, CA, USA) was inserted prior to IMN in 8/12 patients for continuous peri- and postoperative monitoring of indexed cardiac output (CI) and mixed venous oxygen saturation (SvO_2_). Mean pulmonary artery pressures (MPAP), central venous pressures (CVP) and pulmonary capillary wedge pressures (PCWP) were also monitored. In the remaining four patients a pulmonary catheter was not inserted due to unsuccessful procedure (n = 1) or logistical difficulties (n = 3). Indexed systemic (SVRI) and pulmonary vascular resistance (PVRI) and alveolo-arterial oxygen (P_A_O_2 _- P_a_O_2_) differences were calculated using kPa as the unit for gas pressure, the latter by the equation: [(95 × FiO_2_) - (PaCO_2_/0.8)] - PaO_2_. Hemodynamic and lung function parameters were recorded at time points as described in Table 1. The arterial and PA catheters were removed when the patient left the ICU, or at latest on the third postoperative day. Registrations and calculations were performed during IMN and the three following days or for a shorter time period when the patient left the ICU earlier.

**Table 1:** Time schedule for analysis, registrations and calculations

Time	Surgical procedure	Blood sample analyses	Cardiopulmonary registrations and calculations
**A**	Hospital admission	Blood gases, Hb, cytokines, coagulation, fibrinolysis, complement	
**B**	Skin incision	Blood gases, Hb, cytokines, coagulation, fibrinolysis, complement	x
**C**	After nail insertion	Blood gases, coagulation, fibrinolysis, complement	x
**D**	30 minutes after nail insertion	Blood gases, cytokines, coagulation, fibrinolysis, complement	x
**E**	2 hours after nail insertion	Blood gases, Hb, cytokines, coagulation, fibrinolysis, complement	x
**F**	6 hours after nail insertion	Blood gases, Hb, cytokines, coagulation, fibrinolysis, complement	x
**G1**	1. postoperative day at 0800	Blood gases, Hb, cytokines, coagulation, fibrinolysis, complement	x
**G2**	1. postoperative day at 1800	Blood gases	x
**H1**	2. postoperative day at 0800	Blood gases, Hb, cytokines, coagulation, fibrinolysis, complement	x
**H2**	2. postoperative day at 1800	Blood gases	x
**I**	3. postoperative day at 0800	Blood gases, Hb, cytokines, coagulation, fibrinolysis, complement	x

The table shows the time schedule for blood sampling and cardiopulmonary registrations and calculations which include cardiac index (CI), indexed systemic (SVRI) and pulmonary vascular resistance (PVRI), heart rate (HR), mean arterial pressure (MAP), central venous pressure (CVP), mean pulmonary artery pressure (MPAP), pulmonary capillary wedge pressure (PCWP), arterial (SaO_2_) and mixed venous oxygen saturation (SvO_2_), and alveolo-arterial oxygen (P_A_O_2 _- P_a_O_2_) differences.

### Surgical procedure

All patients had general anesthesia with the exception of one, who received spinal anesthesia. A standard antegrade technique was used for reamed IMN (Bicut Intramedullary Reamer System, Stryker, Trauma GmbH, Schöhnkirchen, Germany), and the femur was sequentially reamed 1-2 mm greater than the applied nail diameter. In 11/12 patients a T2 nail (Stryker) was used. In one patient a Groβe-Kempf nail (Stryker) was used. Cephalotine 2 g × 3-4 (Keflin^®^, 50 mg/ml, Lilly, Florence, Italy) was given intravenously as antibiotic prophylaxis according to the hospital routines.

### Blood sampling

Hemoglobin (Hb) was measured in peripheral blood. Arterial- and mixed venous blood gas analyzes were collected according to Table 1. Serial blood samples were collected for determination of coagulation-, fibrinolytic-, complement- and cytokine activation in arterial (sample time A-I) and mixed venous (sample time B-I) blood (Table 1). The samples were collected in Stabylite^® ^(Biopool AB, Umeå, Sweden) tubes, and vacutainer tubes containing ethylenediaminetetraacetic acid (K2EDTA) or 1/10 vol 0.13 M trisodium citrate. The tubes were immediately placed on ice and centrifuged within 30 minutes at 2000 g for 12 minutes at 4°C. Plasma was aliquoted, transferred to 1.5 mL polypropylene tubes, and stored at -70°C until assayed. The samples were thawed only once.

### Assays

Citrated plasma was used for the determination of thrombin-antithrombin-complexes (TAT) (Enzygnost TAT Micro, Behringwerke AG, Marburg, Germany), plasminogen activator inhibitor (PAI-1) (TriniLIZE PAI-1 activity, Trinity Biotech, Jamestown, NY, USA), tissue plasminogen activator (t-PA) antigen (TriniLIZE t-PA antigen, Trinity Biotech), soluble tissue factor (sTF) (Imubind TF Elisa, American Diagnostica Inc., Greenwich, CT, USA) and terminal SC5b-9 complement complex (TCC) (by using an enzyme-linked immunosorbent assay (ELISA) as described by Mollnes et al. [24]). Stabylite plasma was used for determination of t-PA activity (TriniLIZE t-PA activity, Trinity Biotech). When analyzed with the described assays the median plasma levels in healthy humans are; t-PA activity 0.2-2 IU/ml, t-PA antigen 4.0 (women) and 5.5 (men) ng/ml (age 25 - 34), PAI-1 activity 2.60 IU/ml, the normal reference range for TAT complex is 1.0 - 8.0 ug/L, and sTF is normally not present at measurable levels.

The following cytokines were analyzed in EDTA plasma by using commercially available ELISA kits; TNF-α (R&D Systems Europe, Abingdon, UK, Human TNF-α QuantiGlo Chemiluminescent Sandwich ELISA), IL-6 (R&D, Human IL-6 QuantiGlo Chemiluminescent Sandwich ELISA), IL-1β (R&D, Human IL-1β QuantiGlo Chemiluminescent Sandwich ELISA), IL-8 (R&D, Human CXCL8/IL-8 Quantikine colorimetric Sandwich ELISA) and IL-10 (R&D, Human IL-10 QuantiGlo Chemiluminescent Sandwich ELISA). Samples from apparently healthy volunteers evaluated with the applied assays demonstrated the following mean levels; TNF-α 2.88 pg/mL, IL-6 1.32 pg/mL, and IL-10 7.15 pg/mL.

### Statistics

Statistical analyzes were performed using the Statistical Package for Social Science (SPSS) software, version 16.0 (SPSS Inc, Chicago, IL, USA). Normally distributed data are presented as group means and standard error of the mean (S.E.M.). Paired-samples t-test was used for evaluation of increasing or decreasing levels between two measuring points. Non-parametric statistics were used when Kolmogorov-Smirnov test and histograms demonstrated not normally distributed data, as shown for RBCT, coagulation-, fibrinolysis- and cytokine activation. Median levels are then presented. Wilcoxon Signed Rank Test was used as the non-parametric test describing increasing and decreasing levels between two measuring points. Differences were considered significant at P levels ≤ 0.05.

## Results

**Figure 1:**
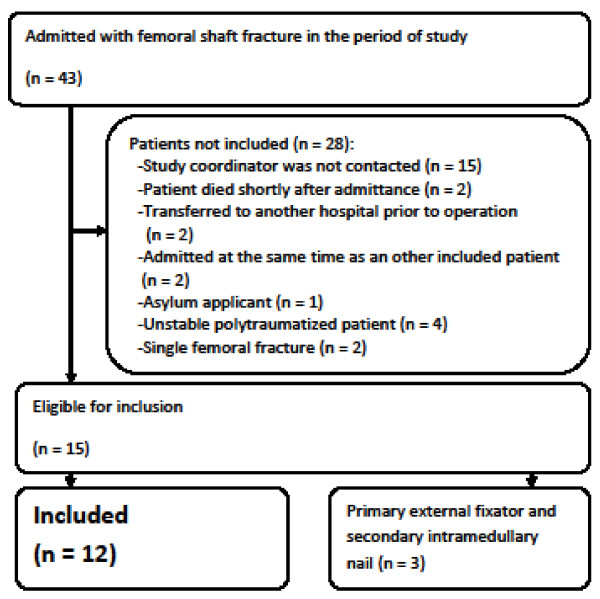
**Flow chart of patients admitted with femoral shaft fracture and included in the study**.

An overview of the patients and the inclusion process is given in Figure 1. Three patients were initially externally fixated and followed the detailed sampling and monitoring program as for the immediately intramedullary nailed patients, but due to the low numbers of patients they were not used as a reference.

12 patients were included, 11 men and 1 woman, aged 27.6 ± 2.5 (range 18-44) years. The mechanisms of injury were car accident (10/12) and fall from heights (2/12). ISS was 31 ± 2.6, NISS was 34.3 ± 2.1, and AIS thorax was 3.7 ± 0.2. The time from injury to admission was 95 ± 11 (range 22 - 365) minutes. One patient had bilateral femoral shaft fractures. The fractures were classified according to AO; 3 A-fractures, 3 B-fractures and 7 C-type fractures. Two fractures were open (Gustilo Anderson 2 and 3A), and in 3 cases the fracture was openly reduced. All included patients had additional extremity- and thoracic injuries, 50% (6/12) abdominal, and 42% (5/12) had head injuries. The time from injury to femoral IMN was 544 ± 42 (range 330 - 830) minutes. The IMN operating time was 106 ± 7 (range 60 - 155) minutes. Number of days in the intensive care unit (ICU) was 16 ± 4 (range 4 - 48). The postoperative course was prolonged by pneumonia (7/12), ALI (3/12), ARDS (3/12) and sepsis (3/12). All except one patient fulfilled the SIRS criteria.

None of the patients received blood product transfusions prior to hospital admission. Hemoglobin level at admission was 12.0 ± 0.7 and at skin incision 9.6 ± 0.4 g/100 mL (p = 0.015). The body core temperature at admission was 36.4 ± 0.3. The variation of administrated blood products was wide, and the majority of transfusions were given between hospital admission and the first day post IMN (Table 2).

**Table 2:** Blood product transfusions.

Time	RBCT	TT	PT
**Admission - IMN (A-B)**	900 (0-4800)	0 (0-500)	0 (0-400)
**IMN - 6 hours post IMN (B-F)**	750 (0-2100)	0 (0-500)	0 (0-400)
**Admission - 1. postoperative day (A-G1)**	2250 (0-6900)	0 (0-1000)	0 (0-1400)
**1. - 2. postoperative day (G1 - H1)**	600 (0-1500)	0 (0)	0 (0-400)
**2. - 3. postoperative day (H1 - I)**	300 (0-600)	0 (0-500)	0 (0-400)
**TOTAL**	**3000 (0-8100)**	**0 (0-1500)**	**200 (0-1400)**

Table 2. The table gives a summary of administrated red blood cell transfusion (RBCT), thrombocyte transfusion (TT), blood plasma transfusion (PT) in milliliter (median and range) from hospital admission to intramedullary nailing (IMN), from IMN to 6 hours after IMN, from hospital admission to the first postoperative day, from the first to the second postoperative day, from the second to the third postoperative day and total transfusions in the patients primary nailed.

### Coagulation and fibrinolysis

In all patients a marked activation of the coagulation- and fibrinolytic systems was seen at hospital admission, but no significant procedure-related effect could be detected.

**Figure 2:**
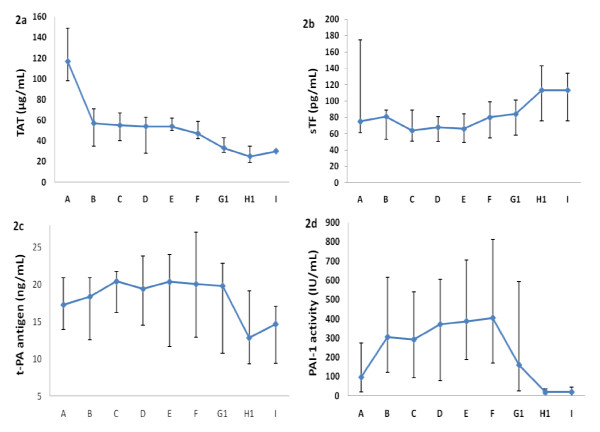
**The figure shows the arterial thrombin-antithrombin-complex (TAT) (2a), soluble tissure factor (sTF) (2b), tissue plasminogen activator (t-PA) antigen (2c) and plasminogen activator inhibitor (PAI-1) activity (2d) (median and 25/75 percentiles) at admission (A), skin incision (B), after nail insertion (C) and 30 minutes (D), two (E) and six (F) hours, the first (G1), second (H1) and third (I) day after nail insertion**.

Arterial TAT (Figure 2a) levels were highest at hospital admission and decreased during the study period. The decrease was significant from admission to skin incision (p = 0.003), and from admission to the third postoperative day (p = 0.007). No increase of arterial TAT plasma concentration related to the IMN procedure was observed.

sTF is normally not present at measurable levels in the circulation. Arterial sTF (Figure 2b) levels were increased at hospital admission and increased during the study period. The increase reached significance from nail insertion (C) to 72 hours (I) post nail insertion (p = 0.011). The highest sTF levels were present at the second and third postoperative day.

The arterial t-PA activity (data not shown) levels were low at admission and stayed low during the study period, and the arterial t-PA antigen (Figure 2c) levels were high at admission and stayed elevated until decreasing from day one post IMN.

Arterial PAI-1(Figure 2d) levels were high at admission and increased further significantly from admission (A) to skin incision (B) (p = 0.001) and from nail insertion (C) to six hours (F) post nail insertion (p = 0.012). From six (F) to 48 hours (H1) post nail insertion (p = 0.005) the PAI-1 activity levels decreased. Peak PAI-1 activity levels were demonstrated at skin incision (B) and at six hours post IMN (F). A procedure-related effect participating in the PAI-1 peak level response could not be ruled out.

No significant differences were demonstrated between arterial and mixed venous blood levels for TAT, sTF, t-PA activity or PAI-1 (data not shown).

Coagulation and fibrinolysis in patients that were primary external fixated demonstrated similar levels as related to IMN. At secondary IMN a procedure-related increase of TAT, sTF and t-PA activity and antigen were present and the PAI-1 response was almost absent.

### Complement activation

Arterial TCC levels were studied in 6 patients. The TCC levels were slightly increased in most patients at admission and increased further (ns) from hospital admission (A) to the third postoperative day (I) (data not shown).

### Cytokines

No significant differences were demonstrated between arterial and mixed venous blood levels for TNF-α, IL-6, IL-10, IL-1β, or IL-8.

**Figure 3:**
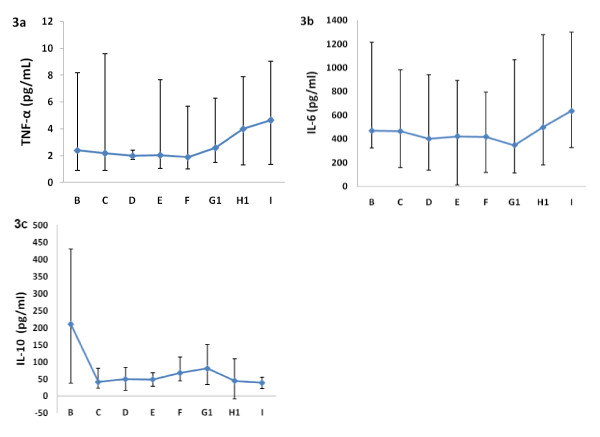
**The figure shows the arterial tumor necrosis factor-alpha (TNF-α) (3a), interleukin-6 (IL-6) (3b) and interleukin-10 (IL-10) (3c) (median and 25/75 percentiles) at admission (A), skin incision (B), 30 minutes (D), two (E) and six (F) hours, the first (G1), second (H1) and third (I) day after nail insertion**.

The arterial TNF-α level remained steady and almost at normal levels from hospital admission to six hours after IMN (F) and then increased significantly to the third post IMN day (I) (p = 0.036) (Figure 3a). The highest level was present the third post IMN day.

Arterial IL-6 levels were elevated already at hospital admission, and for the whole study period the IL-6 levels exceeded 200 pg/mL. Peak levels were observed the third postoperative day (Figure 3b). The increase from skin incision (B) to peak level at the third day post procedure was not significant (p = 0.39).

Arterial IL-10 levels were elevated at hospital admission. A decrease from hospital admission to skin incision (p = 0.009) and a non-significant increase from skin incision to the first post IMN day (p = 0.28) was observed (Figure 3c).

IL-1β levels at all sampling locations were mostly below the lowest detectable level at 0.4 pg/mL (data not shown). IL-8 levels demonstrated no significant increases or decreases within the study group (data not shown).

Cytokine release in patients that were primary external fixated and secondary nailed were both significantly lower and peak levels occurred earlier when compared to the response after initial IMN.

### Cardiopulmonary function

**Figure 4:**
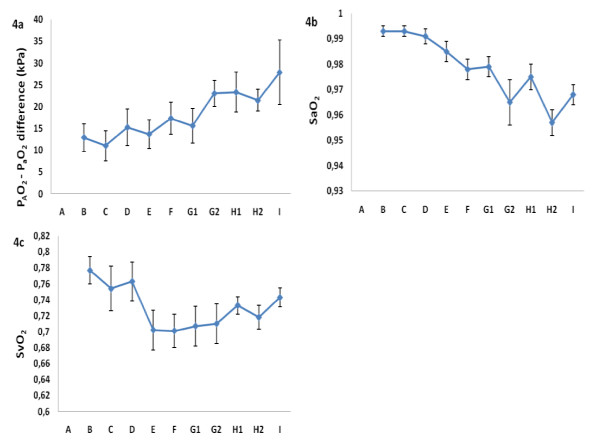
**The figure shows alveolo-arterial oxygen (PAO2 - PaO2) difference (Figure 4a), arterial (SaO2) (Figure 4b) and mixed venous (SvO2) saturation (Figure 4c) (mean ± S.E.M.) at skin incision (B), after nail insertion (C) and 30 minutes (D), two (E) and six (F) hours, the first (G1 and G2), second (H1 and H2) and third (I) day after nail insertion**.

The P_A_O_2 _- P_a_O_2 _difference (Figure 4a) demonstrated elevated and further increasing levels (ns) from skin incision (B) to the third postoperative day (I). Procedure-related increased levels could not be demonstrated. Significantly decreasing SaO_2 _levels (Figure 4b) from skin incision (B) to the third day post IMN (I) (p = 0.000) were observed. SvO_2 _(Figure 4c) decreased significantly from skin incision (B) to two hours after IMN (E) (p = 0.046).

**Figure 5:**
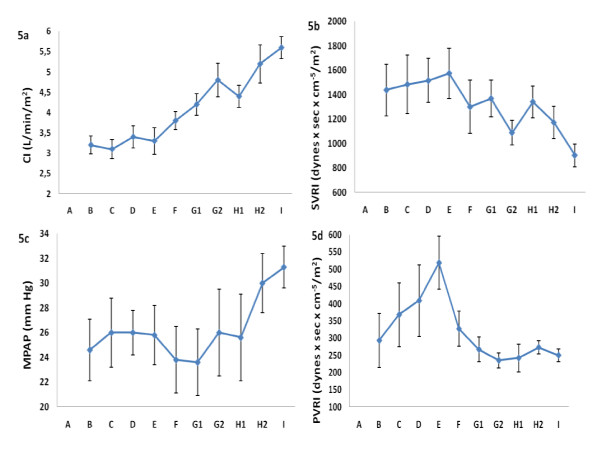
**The figure shows the time course (mean ± S. E. M.) for cardiac index (CI) (Figure 5a), indexed systemic vascular resistance (SVRI) (Figure 5b), mean pulmonary artery pressure (MPAP) (Figure 5c) and indexed pulmonary vascular resistance (PVRI) (Figure 5d) at admission (A), skin incision (B), after nail insertion (C) and 30 minutes (D), two (E) and six (F) hours, the first- (G1 and G2), second- (H1 and H2) and third (I) day after primary IMN**.

CI (Figure 5a) increased significantly from skin incision (B) to the third postoperative day (I) (p = 0.000). MAP demonstrated no significant changes during the study period (data not shown).

The SVRI (Figure 5b) levels were lower than normal at skin incision and the levels, with the exception of a minor procedure-related (ns) increase, continued to decrease (ns) during the study period. The changes in the filling pressures in the right (CVP) and the left (PCWP) side of the heart were modest, but with levels in the upper range of normal levels (data not shown). Both CVP and PCWP demonstrated peak levels at 30 minutes post IMN (D), the increases, however, were not significant. The MPAP (Figure 5c) levels were high at skin incision (B), had a minor transient procedure-related increase, and increased further from the first (G1) to the third postoperative day (I) (ns, p = 0.07). The PVRI levels (Figure 5d) were high at skin incision (B) and increased (ns, p = 0.057) further from skin incision (B) until peaking at 2 hours after the nail was inserted (E).

### Cardiopulmonary function in patients operated with external fixation and secondary nailing

P_A_O_2 _- P_a_O_2 _difference after external fixation was comparable to the levels after primary IMN. Related to secondary nailing, the P_A_O_2 _- P_a_O_2 _difference was elevated at skin incision, and demonstrated decreasing levels during the study period. The SaO_2 _levels after external fixation and secondary nailing were comparable to the levels after primary IMN. Minor increases of PVRI, MPAP and PCWP values were seen between 30 minutes and two hours after secondary IMN.

## Discussion

In this study on 12 polytraumatized patients with femoral shaft fractures we investigated the impact of IMN on cardiopulmonary function and inflammation- and coagulation/fibrinolytic responses. The main observation was that an additional effect of the primary IMN, a second hit, on the activation of coagulation and fibrinolysis could not be clearly identified. A transient procedure-related PVRI increase was present. This may be a result of intravasation of intramedullary content. Delayed peak levels of arterial TNF-α, IL-6 and IL-10 levels were observed and might be the result of a second hit phenomenon. Primary IMN did not demonstrate a negative impact on pulmonary shunting.

In the present study systemic activation of the coagulation system, represented by arterial TAT level increase, was present at patient admission to the hospital. The high TAT levels were mainly related to the injury per se, and primary IMN did not result in additional TAT generation. Activation of fibrinolysis, measured as elevation of t-PA activity, was low. However, the t-PA antigen levels were high at hospital admission and stayed high before it dropped at 48 hours after IMN, which verify an increased fibrinolytic activity for much longer than the evaluation of t-PA activity alone suggests. The inhibition of the fibrinolysis, measured as augmented PAI-1 activity levels, was present at admission, and increased further. PAI-1 is produced upon stimulation by endothelial cells, platelets, fibroblasts and smooth muscle cells and functions as an acute phase reactant [25], and also binds circulating t-PA rapidly. Despite the prolonged increase in t-PA antigen levels, the functional effect of the PAI-1 increase (which is faster than other acute phase reactants like CRP and fibrinogen), combined with the nearly absent t-PA activity increase (neutralized by PAI-1) definitely represent a fibrinolytic shutdown and a prothrombotic state, which is often prevailing after orthopedic surgery or trauma.

Tissue factor (TF) is the most potent trigger of the coagulation system known [26]. sTF is normally not present at measurable levels in the circulation [27]. Marked peaks of plasma TF activity have been demonstrated during bone preparation in total hip replacement surgery [28], which indicates release of TF-rich material to the systemic circulation from traumatized tissues, mainly the bone marrow which is a rich source of TF. This mechanism may also explain the sTF levels found in the present study; elevated arterial sTF blood levels were demonstrated at hospital admission (A), which decreased slightly until skin incision (B) and then increased significantly until the third postoperative day (I), indicating an enduring procoagulant state.

Generation of proinflammatory cytokines has been demonstrated to be proportional to the extent of tissue injury and hypoxia [29-32]. TNF-α levels in our study were steady and slightly elevated until six hours post procedure (F) and were still increasing at day three post IMN. The still increasing levels at day three are not in agreement with other studies. Spielmann et al. [33] demonstrated the highest TNF-α level in trauma patients 12 hours post injury, Kobbe et al. [34], however, demonstrated no significant elevation of TNF-α. Great variations in TNF-α levels (when measured) are observed in clinical studies, which can be explained by the short half-life of TNF-α [34].

The IL-6 levels in the present study were in general higher than 200 pg/mL, a level which is associated with SIRS [30]. In the one patient without SIRS, peak IL-6 level (at 6 hours post procedure) was 106 pg/mL. The elevated IL-6 levels in the present study were in agreement with other investigations demonstrating a post injury association between increased levels of IL-6, and high ISS and postoperative complications [29-32]. The IL-6 level peak in the present study occurred later than peak levels observed in other studies [13,14,29,31,35] in which IL-6 peaked 4 - 24 hours post injury or post procedure and persisted for 3 - 10 days [31,35]. Orthopedic surgery has in particular been associated with local release of IL-6 [36]. Pape et al. [14] investigated patients with femoral shaft fractures and found venous IL-6 peak levels 24 hours after IMN. When the femoral fracture was initially stabilized with external fixation and secondarily converted to an intramedullary implant, they did not, however, observe a surgery-related IL-6 increase [14]. These results are inconsistent with the results in the present study demonstrating arterial IL-6 peak level at the second postoperative day after primary IMN and also a procedure-related IL-6 response after secondary IMN. The IL-6 levels after secondary nailing in our study were significantly lower than the levels after primary IMN. The results from the study of Pape et al. [14] were also inconsistent with the results from the study of Morley et al. [37] in which blood samples from the femoral canal before and after reaming of the canal showed very high levels of IL-6 after intramedullary reaming (median 3947 at opening and 15903 pg/mL after reaming). These very high levels indicated a significant local inflammatory reaction following the dual trauma (fracture and IMN). Due to intravasation and pulmonal sequestering of bone marrow content at intramedullary pressure increase during femoral canal reaming, an additional pulmonary IL-6 activation has been suggested in an experimental porcine study [38]. Levels of IL-6 in both mixed venous and arterial blood were analyzed in the present study, but the results did not confirm the suggestion.

No IL-1β or IL-8 response was observed in the present study. This is consistent with other studies [34,38,39].

For the evaluation of the anti-inflammatory response IL-10 was studied. IL-10 levels were elevated during the study period, and peak IL-10 level was observed at the first day post primary IMN. This IL-10 response was delayed compared to other studies demonstrating an IL-10 response after 1 - 6 hours post injury [34,40].

In this study we have used the non-parametric tests for evaluation of coagulation-, fibrinolysis- and cytokine activation. Both effect size and power calculations of the results have demonstrated large variations, which indicate a possibility for a Type 2 error and insignificant results due to the limited sample size.

In the literature, there is no uniform understanding of the effect of IMN on hemodynamics and pulmonary function. Early operative fracture treatment, especially of large long bones (femur), has empirically been associated with reduction in the occurrence of pulmonary failure (ARDS) [2,41,42]. However, some data also indicate that the early internal stabilization of these fractures in itself can have negative impacts on pulmonary function. The total effect of IMN on morbidity seems, however, to be beneficial.

The reported incidences of ALI and ARDS in severely injured patients with femoral shaft fractures are not uniform [2,43,44]; a higher incidence is suggested if the fractures are accompanied by thoracic injuries [43]. In the present study 6/12 patients had ALI or ARDS (3/12 ALI and 3/12 ARDS), and all the patients had thoracic injury. The pathophysiology of the early ALI after trauma is fluid leakage and pulmonary edema combined with inflammatory cell infiltration of pulmonary tissue. In addition, a hypoxemic vasoconstriction and capillary microthrombosis creates a ventilation-perfusion mismatch, clinically manifested as hypoxemia. The P_A_O_2 _- P_a_O_2 _difference increased in the severely injured patients in this study as a surrogate indicator for increased pulmonary shunting. Pulmonary vascular changes may cause an increased pulmonary vascular resistance and increase the MPAP [45]. In the present study MPAP was elevated already at skin incision, and demonstrated a minor procedure-related increase, before continuously increasing towards the end of the study period. Simultaneously, a procedure-related PVRI level increase was observed. In previous human studies increased MPAP, PVR and CVP plus decreased PaO_2 _have been associated with pulmonary embolism [46]. These associations, however, have to be interpreted carefully as pressure increases in previous normal, easily distendable, pulmonary blood vessels may retain undetectable until at least 30% of the vessels are occluded [47]. In pre-injured lungs the effect of compensatory dilatation will not occur equivalently, and the flow in the still open pulmonary vessels will increase and create significantly elevated PVR levels. The filling pressures of the heart, CVP and PCWP, are influenced by treatment, quantum of intravenous fluid/blood administration and the titration of ventilator volumes, pressures and rates. As strict control of these parameters is impossible in a clinical setting, CVP and PCWP are not suitable for the evaluation of impact of surgical interventions on cardiac function.

Polytraumatized patients usually consist of a heterogeneous group of patients, and firm conclusions from clinical studies are difficult to draw. Differences in the extent of injury, time from injury to hospital admission, time from admission to operation, extent of further operations and amount of blood product transfusions induce a wide range of variance. The included patients in the present study were extensively examined, which also made the patient inclusion and follow-up challenging. The present patient material consisted of patients with high ISS, femoral shaft fractures equally treated, and all registrations, calculations and analyzes were strictly related to IMN. However, the included patients were relatively few. We did not differentiate and relate the observations and calculations to specific organ system injuries. For such differentiation, larger studies and multi-centre trials are needed.

## Conclusions

In the severely injured patients with femoral shaft fractures operated with primary IMN we observed a substantial response related to the initial trauma. We could not demonstrate any major additional IMN-related impact on the inflammation or the cardiopulmonary function parameters. A transient cardiopulmonary response related to IMN, however, was demonstrated. Delayed arterial TNF-α, IL-6 and IL10 peak levels were observed, and it could be questioned whether these changes were related to the procedure. These results have to be interpreted carefully due to the relatively few patients included in the study. The inflammation-associated complication rate was high.

## Abbreviations

AIS: Abbreviated Injury Scale; ALI: Acute lung injury; ARDS: Adult respiratory distress syndrome; CI: Indexed cardiac output; CVP: Central venous pressure; EDTA: Ethylenediaminetetraacetic acid; Hb: Hemoglobin; ICU: Intensive care unit; IL: Interleukin; IMN: Intramedullary nailing; ISS: Injury severity score; MAP: Mean arterial pressure; MOF: Multi Organ Failure; MPAP: Mean pulmonary artery pressure; NISS: New injury severity score; Ns: not significant; PA: Pulmonary artery; PAI-1: Plasminogen activator inhibitor-1; PaO_2_: Partial pressure of arterial oxygen; P_A_O_2_-P_a_O_2_: Alveolo-arterial oxygen difference; PCWP: Pulmonary capillary wedge pressure; PVR: Pulmonary vascular resistance; PVRI: Indexed pulmonary vascular resistance; RBCT: Red blood cell transfusion; S.E.M.: Standard error of the mean; SIRS: Systemic inflammatory response syndrome; SPSS: Statistical Package for Social Science; sTF: Soluble tissue factor; SvO_2_: Mixed venous oxygen saturation; SVR: Systemic vascular resistance; SVRI: Indexed systemic vascular resistance; TAT: Thrombin-antithrombin complex; TCC: Terminal complement complex; TF: Tissue factor; TNF-α: Tumor necrosis factor-alpha; t-PA: Tissue plasminogen activator.

## Competing interests

Each of the authors certifies that he or she has no commercial associations that might pose a conflict of interest in connection with the submitted manuscript. The manuscript has not been submitted or published elsewhere.

## Authors' contributions

EEH, TL, HO, TA, RØS, JEM and OR designed the study or interpreted the evidence it presents, EEH gathered the data, EEH and RØS analyzed the data, TA and Lisbeth Sætre analyzed the blood samples, EEH wrote the initial draft, and EEH, TL, HO, TA, RØS, JEM and OR ensured the accuracy of the data and analysis, read and approved the final manuscript.
